# Dealing with difficult choices: a qualitative study of experiences and consequences of moral challenges among disaster healthcare responders

**DOI:** 10.1186/s13031-022-00456-y

**Published:** 2022-05-08

**Authors:** Martina E. Gustavsson, Niklas Juth, Filip K. Arnberg, Johan von Schreeb

**Affiliations:** 1 Department of Global Public Health, Centre for Research on Healthcare in Disasters, Health Systems and Policy (HSP), Karolinska Institutet, Stockholm, Sweden; 2 Department of Learning, Informatics, Management and Ethics (LIME), Stockholm Centre for Healthcare Ethics (CHE), Karolinska Institutet, Stockholm, Sweden; 3 Department of Medical Sciences, National Centre for Disaster Psychiatry, Uppsala University, Uppsala, Sweden

**Keywords:** Disaster, Moral challenges, Moral stress, Moral distress, Disaster responders, Humanitarian workers

## Abstract

**Background:**

Disasters are chaotic events with healthcare needs that overwhelm available capacities. Disaster healthcare responders must make difficult and swift choices, e.g., regarding who and what to prioritize. Responders dealing with such challenging choices are exposed to moral stress that might develop into moral distress and affect their wellbeing. We aimed to explore how deployed international disaster healthcare responders perceive, manage and are affected by moral challenges.

**Methods:**

Focus groups discussions were conducted with 12 participants which were Swedish nurses and physicians with international disaster healthcare experience from three agencies. The transcribed discussions were analyzed using content analysis.

**Results:**

We identified five interlinked themes on what influenced perceptions of moral challenges; and how these challenges were managed and affected responders’ wellbeing during and after the response. The themes were: “type of difficult situation”, “managing difficult situations”, “tools and support”, “engagement as a protective factor”, and “work environment stressors as a risk factor. Moral challenges were described as inevitable and predominant when working in disaster settings. The responders felt that their wellbeing was negatively affected depending on the type and length of their stay and further; severity, repetitiveness of encounters, and duration of the morally challenging situations. Responders had to be creative and constructive in resolving and finding their own support in such situations, as formal support was often either lacking or not considered appropriate.

**Conclusion:**

The participating disaster healthcare responders were self-taught to cope with both moral challenges and moral distress. We found that the difficult experiences also had perceived positive effects such as personal and professional growth and a changed worldview, although at a personal cost. Support considered useful was foremost collegial support, while psychosocial support after deployment was considered useful provided that this person had knowledge of the working conditions and/or similar experiences. Our findings may be used to inform organizations’ support structures for responders before, during and after deployment.

**Supplementary Information:**

The online version contains supplementary material available at 10.1186/s13031-022-00456-y.

## Background

A disaster can be seen as a breakdown of a society’s normal functioning due to hazardous events that cause material, human, environmental or economic losses [1]. In disasters capacities and resources can be insufficient due to overwhelming needs and then external assistance is essential for the affected community to cope [1, 2]. In disaster settings, deployed disaster healthcare responders are faced with significant challenges. They carry out their work under extreme pressure and need to make prompt decisions in often dangerous and challenging contexts. The scarcity of resources such as time, equipment, and capacity hinder the provision of optimal care to all; resources must be strictly prioritized [2]. The choices involved in deciding who and what to prioritize expose responders to moral challenges well beyond those encountered during normal healthcare provision. Moral stress is a reaction to a moral challenge, a moral stressor in which the health care worker cannot act in accordance with his or her own moral values. Unresolved moral stress may lead to long-term moral distress, which is a negative stress reaction that in turn could lead to secondary psychological consequences [3].

### Moral distress in disaster contexts

Research of moral distress within normal health care settings has developed from Jameton’s original definition of moral distress as something that arises *“when one knows the right thing to do, but external constrains make it nearly impossible to pursue the right cause of action”* [4]. Within normal health care settings morally challenging situations such as perceived futility of care, burdensome technology and delayed end-of-life care has been reported as contributing to moral distress [5]. However, disaster healthcare responders are a particularly vulnerable group, as they often work in unsafe contexts with overwhelming workloads [6]. Available professional ethical guidelines are of limited use because they do not sufficiently capture the complexity of disasters nor the pressure that responders are exposed to [7]. A high probability of exposure to moral challenges, potential traumatic events, and other work-related stressors entails a risk that disaster healthcare workers will experience traumatic stress, burnout and moral distress [8–10]. Preparedness and training for the work environment has been stated to increase rescuers’ resilience regarding managing ethically difficult situations [11, 12]. Further, professional collaboration and a positive ethical culture are reported to be protective factors for moral distress within normal health care [13–16]. Risk- and protective factors which affects the development of moral distress, and its consequences has been illustrated in a “Conceptual model of moral distress and its consequences” by Gustavsson, Arnberg, Juth and von Schreeb, 2020. In this model, moral residue (lingering moral distress) is one of the secondary consequences that in turn might lead to burnout. This model will be used as a framework to better understand the risk- and protective factors that influence disaster health care responders’ wellbeing and will be further populated [3].

### Consequences of moral distress

Unresolved moral challenges are reported to lead to negative consequences for the wellbeing of responders such as burnout and depression, and these moral stressors are in addition to other common stress factors [17]. This was highlighted after the Ebola outbreak in 2014–2015, when more cost-effective preventive public health measures were prioritized over individual clinical care in order to maximize efforts to stop the epidemic. The responders were exposed to moral challenges when they instead had to prioritize populations over individual patient care [18, 19]. Similar challenges have been reported during the ongoing COVID-19 pandemic, highlighting an acute need for effective support strategies for strained healthcare workers facing moral stress [20–22]. Moral injury (acting or failing to prevent and act which violates own moral values) is another related concept to moral distress which has expanded during the COVID-19 pandemic, as health care workers has been prone to more potentially traumatic moral events [23–25].

Although several alarming reports have emphasized the negative consequences of moral distress among responders, the burden of moral distress within this population is still poorly understood [26]. Psychological distress and burnout are reported as frequent consequences among responders and affect not only individuals but also the quality of care that they provide [27]. Furthermore, sick leave and high dropout rates among healthcare personnel result in high costs for society [26, 28]. The existing literature highlight the need for responders to be well equipped and mentally prepared to confront moral challenges in disasters. Improved disaster preparedness through training and education for emergency and disaster personnel has been emphasized by organizations as well as by the Swedish government [29, 30]. The links between moral stress, moral distress, and wellbeing have been scarcely studied among disaster healthcare responders (henceforth called responders) [31]. In order to minimize the negative effects of moral distress, which would be of importance for both individual responders and organizations, there is a need to investigate the causes of morally challenging situations, and beneficial preparations and support mechanisms. To optimally prepare and support responders and their work, more knowledge is needed about the type and extent of moral challenges they experience and what actions they take to manage and mitigate the negative consequences of these challenges.

## Methods

### Aim

The aim of this study is to explore how disaster healthcare responders perceive and manage moral challenges and how they perceive these moral challenges in relation to their wellbeing.

Focus group discussions (FGDs) were conducted with Swedish disaster healthcare responders with experience from international disaster response. This method was chosen because it can provide valuable insight into a subject about which a targeted, experienced group collectively understands and reasons. The discussions, through stimulated interaction, can provide large variation in the different aspects and perspectives, which may not be the case when using individual interviews [32, 33].

In this study we use the term humanitarian workers to categorize responders from non-governmental humanitarian organizations, while disaster healthcare responders include both humanitarian and governmental responders in disasters.

### Recruitment

Three Swedish organizations with extensive experience in disaster and humanitarian response, namely, the Swedish Red Cross (SRC), Medécins sans Frontières (MSF), and the Swedish Civil Contingencies Agency (MSB), were contacted and introduced to the aim of the study. SRC and MSF are non-governmental organizations guided by humanitarian principles, whereas MSB is a governmental agency that works with and provides support to organizations during and after disasters. The latter organization provides salary-based compensation, SRC provides fluctuating salary-based compensation depending on the type of contract, and MSF offers voluntary-based compensation to responders.

Two of the organizations provided the study team with a list of participants that the organization had checked for eligibility and asked about their willingness to take part in the study. One organization provided contact details to eligible participants, whom the study team then approached about their willingness to participate.

### Procedure

Potential participants were contacted by email and telephone and provided with information about the study. Those who fulfilled the criteria for participation provided informed consent and received more detailed information about the topic of the discussion in order to facilitate their preparedness for the topic. They were encouraged to think of a morally challenging situation, described as “a difficult situation”, in their professional role during their response, during which they could not do what they felt was the morally right thing to do, resulting in a negative reaction, such as frustration or hopelessness. Two eligible participants declined participation at this point, as they felt that they had no such experiences to contribute. The remaining participants were able to choose available dates for the FGD from a set of options. Four participants were unable to attend any FGD and were excluded from the study.

First, a pilot group discussion was conducted to try out the interview guide and the question format. The guide and question format were then rearranged to better fit the flow of the discussion, although no questions were changed. The data from the first group were included in the analysis, additional to the three FG. Three groups were then assembled, and participants were selected for each to obtain groups that were heterogeneous in terms of sex, age, experience and background, with mixed group members to capture broader variation in the discussion of various experiences.

If participants reacted with especially strong emotions during the FGD, they would be offered a telephone consultation with a clinical psychologist from the study team. However, no participant felt the need for such a consultation. Ethical permission was obtained for this study from the Regional Ethics Review Board, Stockholm (2017-12-21, DNR: 2017/2182-31).

### Participants

Eligible participants had to be healthcare professionals with experience of being deployed to a disaster or humanitarian emergency at least once within the past five years. Health care professionals were excluded if they had repatriated from a mission within the past six months, to allow them time to settle down. The study also excluded individuals who were willing to participate in the FGD but unable to attend on the proposed dates due to a scheduled deployment before the FGD.

The age of the 12 participants ranged from 35 to 64 years (mean age: 48); there were eight women and four men. Five of the participants were physicians, some of whom specialized. Seven participants were nurses, and some specialized. Participants’ working experience within their profession ranged from nine to 37 years. The number of international response missions varied from one to seven, and the total length in the field ranged from two months to four years. Six participants worked with MSF, two with MSB, and two with RC; two had worked with two of the organizations. Five participants had additional experience from other types of international fieldwork not related to a disaster or emergency response, such as projects focusing on neglected diseases. Participants had experience from response work in natural disasters (earthquake, flooding), outbreaks (Ebola, cholera), man-made disasters (conflicts, protracted emergencies, refugee crisis and rescue operations in the Mediterranean), see Table 1. Experiences from specific types of response were not considered when assembling the FG, since the aim was to explore their perception and management of moral challenges in disaster response.

**Table 1:** Background characteristics

Characteristics	Occupation and gender				Total
	Nurses (females)	Nurses (males)	Physicians (females)	Physicians (males)	
Number	5	2	3	2	12
Age (years)	35–57	44–57	37–42	63–64	35–64 (48 mean age)
Work experience in profession (years)	9–30	10–34	10–11	35–37	9–37
Number of international missions in total^a^ (number)	2–7	2–5	1–3	3–6	2–6
Number of emergency/disaster response (number)	2–5	2–5	1–2	3	1–5
Total time within international missions^a^ (months)	5–52	2–5	6–30	6–45	2–45
Countries/areas of international missions^a^ (alphabetical order)	Afghanistan, Angola, Bangladesh, Bosnia, Democratic Republic of Congo, Ethiopia, Guinea, Haiti, Iraq, Jordan, Lebanon, Liberia, Libya, Mediterranean, Mozambique, Niger, Nepal, Palestine, Sierra Leone, South Sudan, South Africa, Sudan, Syria, Tanzania, Uzbekistan, West-Sahara				

^a^Represent international deployments which include both emergency/disaster response and non-emergency response

#### Focus group discussions

An interview guide was developed with an aim to cover the same topics within each group discussion and to ensure the flow of discussion with probes for joint discussions (Additional file 1: Appendix 1). The interview guide was discussed within the research group and with an external person with disaster response experience.

The FGDs were conducted after working hours in Stockholm, Sweden, during April 2018. The discussions were led by the first author, who has experience from international disaster responses. The sessions were audio recorded. An assistant took notes and observed the group and the discussion, ensuring that all themes in the interview guide were covered. First, participants were introduced to each other. Then, the moderator presented the outline of the discussion and distributed a brief questionnaire to collect information about age, profession, year of professional experience, types and number of disaster responses, and countries/context of the disaster response (see Table 1).

The discussions were held in Swedish. The opening question was one regarding a specific difficult situation concerning a moral challenge that participants had been encouraged to reflect upon in the inclusion email or phone call. The following questions elaborated on the details about the causes of the situation, the consequences, how participants managed the situation, how they were professionally and personally affected, the feelings that came with it, and what type of support they received during and after these challenging situations. Questions related to how they managed settling into daily life at home and the level of support they received after repatriation were then asked. The discussions ended with a question about recommendations and advice to future healthcare responders.

The moderator promoted an active discussion encouraging all participants to contribute. The level of interaction within the group varied in each FGD but was collectively stimulated with different individual stories told by participants during the discussion. Participants were able to share information about sensitive topics, and the discussions were perceived by the study team as developing organically. When particularly difficult experiences surfaced, participants expressed gratitude for being able to discuss them with others who had similar experiences. Each FGD lasted approximately two hours.

### Data analysis

The analysis process started after each FGD, when the moderator and the assistant discussed the content and notes from the discussion. The recordings were transcribed verbatim by the first author. The corpus was analysed by means of qualitative content analysis as described by Graneheim and Lundman [34]. The analysis concerned manifest content in the data [35]. First, transcripts were read several times to gain an overview. Each transcript was systematically broken down into meaningful units where words and expressions in the text stating a specific meaning were identified and given a descriptive label. Subcategories were then created by categorizing the meaningful units, condensed into broader categories, and subsequently arranged into themes. Subcategories, categories and themes represent three levels with increased abstraction of information, and themes represent the highest level of abstraction. The analysis was performed using *Nvivo* software. Excerpts that best illustrated each subcategory were translated to English to be reported here. The first author made the first translation of the excerpts, which then were revised after discussion with the co-authors. The total number of chosen excerpts can be found in Additional file 2: Appendix 2. The excerpts are deidentified such that identifying words have been replaced with an “x” or with less detailed information such as “organization x” or “the other area”; “…” indicates a pause, and “//” denotes that irrelevant text has been removed. Words in parentheses have been added to complete or clarify the meaning of the sentence. Excerpts that contain “P1” and “P2” indicate a conversation between persons. The labels of the excerpts indicate from which FGD the quotations came from.

## Results

The analysis resulted in five themes that represent 16 categories, which in turn include a subset of the 81 identified subcategories. The themes are linked, and they influence how the difficult situations causing moral stress were managed and how moral stress affected wellbeing among the participants both during and after the response. The five themes were “type of difficult situation”, “managing difficult situations”, “tools and support”, “engagement as a protective factor” and “work environment stressors as a risk factor”. An overview of the themes, categories and subcategories is displayed in Table 2.

**Table 2:** Result of content analysis from FGD with disaster healthcare responders about moral challenges

Subcategories	Categories	Themes
1. Politics	Not being able to act and being hindered by external circumstances	Type of difficult situation: duration, frequency, and intensity
2. Difficult priorities		
3. Cultural aspects in the work context		
4. Lack of resources and enormous needs		
5. Being hindered by the organization		
6. Not being able to act		
7. Not being able to give care and comfort		
8. Own security hindering giving care		
1. Not standing up for values	Actions perceived as insufficient	
2. Making different priorities		
3. Frustration in not having enough capacities to act sufficiently		
4. Inequalities between you and the population/local colleagues		
5. Balancing collaboration with other partners and authorities		
6. Having to build trust before acting		
7. Frustration due to lack of coordination		
8. Frustration due to lack of leadership		
1. Creating false hope	Imposing harm instead of helping	
2. Causing harm instead of helping		
3. Different motivations and attitudes among colleagues causing bad reputation		
4. Being part of a bad decision/act		
5. Colleagues’ wrongdoing		
6. Being accused of a decision taken		
7. Contributing to/causing inequalities		
1. Constructive solutions	Resolving the situation	Managing difficult situations
2. Focus on solutions rather than problems to adapt to the context		
3. Create collaborations and ask for help to resolve		
4. Using experience		
5. Do something instead of nothing		
6. Sense of teambuilding		
7. Fighting and using courage to resolve		
8. Discontinue the mission		
1. Realizing unforeseen consequences	Consequences of unresolved situations: moral distress	
2. A sense of powerlessness and ruminating		
3. Feeling isolated and surreal		
4. Feeling scarred		
5. Not worth it, leaving earlier		
6. Leaving earlier, to save yourself		
7. Depending on the time in the field		
8. More tough to come home than to be there		
9. Finding a new everyday life		
10. Feeling alienated		
11. Feeling lonely		
12. It eventually hits you		
13. Losing something		
14. Having a pause before going back to work		
1. Contribute to improvements and better support	Positive consequences of managing difficult situations	
2. Creative solutions		
3. Learning from mistakes		
4. Self-confidence and learning things about myself		
5. More aware of inequalities		
6. Growth and being more grateful		
1. Relying on informal support while there	Informal support	Tools and support
2. Creative support-seeking while there		
3. Lack of access to normal social support while there		
4. Lack of social support when home		
5. Creating your own support when home		
6. Not being lonely with the feeling		
1. Lack of accessible formal support from the organization while there	Formal support	
2. Formal support when home (debriefings, psychological support etc.)		
3. Lack of appropriate support when home		
1. Preparations, courses, and trainings	Preparatory support	
2. Briefings and information before		
3. Lack of adequate information before		
1. Other tools used to relieve stress while there	Other tools	
2. Going back to work, or take a pause		
3. Own methods used to resolve/adopt when coming home		
1. Achieve good for others	Altruistic motivation	Engagement as a protective factor
2. Making a difference		
1. Creating learning and experience	Professional and personal motivation	
2. You do what you can		
1. Working within a structure	Framed by the professional role	
2. You must follow guidelines		
1. We have problems in our own health care system	Challenges are part of the work	
2. Have agreed on the conditions		
1. Risk-taking in dangerous environments, security risk	Work environment	Work environment stressors as a risk factor
2. Not being able to go back to normal work directly, financial, and personal health risk		
3. Traumatic events		
1. Malfunctioning team	Stress and exhaustion	
2. Facing continuous suffering		
3. Having a role of responsibility in stressful environments		

### Theme 1, type of difficult situation: duration, frequency, and intensity

This theme comprises three categories that represent the different types of difficult situations that responders face.

Participants discussed the difficult situations with descriptions of frustration, powerlessness, helplessness, anxiety, anger, and sadness when being hindered or unable to appropriately address recognized needs. Some situations were described as resulting in imposed harm or created false hope among patients instead of helping as intended.

#### Not being able to act and being hindered by external obstacles

This category represents eight subcategories that characterize situations in which obstacles hindered participants from acting upon their moral values or acting according to what they perceived was right.

The external circumstances included a lack of resources and enormous needs, political and cultural aspects, organizational decisions, and the need to prioritize one’s own security. Participants expressed frustration and powerlessness when faced with situations in which they could not address needs for different reasons. Being forced to make difficult prioritization decisions leaving individual patients behind or being prevented from providing needed care or comfort were examples of these situations. All participants noted that it was frustrating to be prevented from acting when facing a moral challenge.

FGD 1: Yes, as some kind of ... justification for these types of decisions was like ... which I thought you got ... We talked a lot about (it) and so .... the hard fact was that we were actually there more to fight an epidemic than to help individuals actually.

FGD 2: And it was full ... it was full war ... there were ... so very badly sick people and injured…multi-traumas...there were horrible orthopaedic things because they were, eh. … But ... It became so much so I didn’t know what... I had to do something, I couldn’t really do anything…or really should not. We should only evacuate; we should only be there.

#### Actions perceived as insufficient

This category represents eight subcategories that describe situations in which participants tried their best but nonetheless felt that their actions were not good enough or not consistent with their moral standards.

When participants or their organization could not adhere to important values or needed to prioritize differently than they were used to or when they lacked sufficient means or capacities to act according to necessity, feelings of insufficiency and frustration ensued. The difficulties related to balancing collaboration with authorities and other organizations as well as trying to build trust with authorities and the community were a source of frustration when they led to delays in the response. However, participants stated that functional collaboration was necessary. A lack of coordination and lack of clear leadership communication among organizations within the response and in their own organization made the response ineffective and was a source of frustration for all participants. Further, noticing inequities between themselves and the population they were there to assist or between themselves and local colleagues created feelings of sadness, anger and frustration among several participants.

FGD 1: P2. But I was thinking that when I was there ... my role as a doctor and (to) do exactly the right treatments and things like that got a pretty serious setback so to say...

FGD 3: Because you put higher demands on those that you work with, of course. It is us, or like, we must, someone must ... stand up for what we do. And that I often think makes it more frustrating than the work in itself – if the organization does not support you in what you do or, yes. And not only, not just, like having opinions but also, sort of simply facilitate the work.

FGD 1: …But you are still not prepared anyway, because of the heat and… and to manage like ... // So in a short time, you want to do so much more ... I was a supervisor nurse then ... had 60 patients ... in high risk and ... and had four tents.

#### Imposing harm instead of helping

This category represents seven subcategories concerning situations where participants felt that their actions resulted in harm instead of help.

Several participants expressed that they were very displeased when the response had harmful rather than helpful results. Some participants described situations in which they felt that the response created false hope among the population that they sought to help. Certain participants reported frustration when they realized that decisions or actions taken contributed to increasing inequalities, particularly related to increased disparities between population groups.

Some participants described situations in which motivational conflicts among colleagues became contagious within the team. This was described as diverging motivations or attitudes among colleagues that became apparent in encounters with patients and/or within the team. All participants argued that being part of a bad decision created a feeling of complicity. This feeling extended to situations in which participants felt they were associated with other colleagues’ misbehaviour or intentions. Several participants reported situations in which their colleagues acted against the protocols or acted immorally against patients and local colleagues. Colleagues’ misconduct was reported as especially frustrating by participants because it was an unforeseen challenge. Another unexpected challenge was being accused by colleagues of making a poor decision that had to be made due to the acute situation and therefore it was difficult to manage the colleagues’ reaction in the disaster setting.

FGD 3: And it was a nurse who worked with him who said that yes, unless he leaves, then I leave. And which she later did, she left because she couldn’t work with him. She had written down every day what he had done (wrong): He told the patients this, he prescribed this antibiotic, he did this, and, like, she had documented ... for a month, wrong, wrong, wrong. But, in the end, she gave up, like no, this doesn’t go, I can’t.

FGD 3: And then I experienced, huge ethical dilemmas, when I saw that those who worked there (with us), not at all were humanitarian. It was really hard for me, and I still have a hard time with this... When I work for (organization x) we gave food to all refugees, in a dose that is, a normal food-dose as well ... But there they would receive half an apple and a half sandwich, per day.

FGD1: But we work in healthcare as well, we are used to that kind of people ... like you are unable to manage certain patients within surgical care also ... // ... like here at home ... // ... But it is this thing that you can stand there by yourself, like this decision, like, I ... the example I had. //...That you stand alone with the decision yourself and that you then, by a colleague, can be (accused) like this.

### Theme 2, managing difficult situations

This theme includes three categories about how difficult situations were managed and the consequences of resolved and unresolved situations.

#### Resolving the situation

This category comprises eight subcategories that represent how participants managed and resolved difficult situations. The descriptions of managing difficult situations were mostly focused on constructive, individual solutions. Several participants declared that they tried to focus on solutions rather than problems and that it was necessary to adapt to the current context to better understand the environment and the underlying causes of problems. Some participants expressed that they sometimes had to act, even when they knew it was not the best solution, as a way to manage the situation—to feel that they had at least done something.

All participants believed that creative collaboration with national and international staff was a method to resolve issues, as well as asking for help and support in the situation. Some participants described that they contacted different people outside the response team for advice when confronted with a situation in which they could not find an acceptable solution or act according to their moral values. Doing so was a way for participants to resolve problems when they did not receive adequate support within the team or from the organization. All participants considered collaboration with colleagues to be a useful method to resolve difficult situations and common problems. Resolving issues together resulted in a positive sense of teambuilding. Some participants expressed that they used their earlier experiences as a guide, which was seen as helpful: this strategy made it easier to find constructive solutions.

Many participants mentioned that they sometimes had to be courageous in order to fight and be a voice for the good and to resolve issues. They did so despite feeling a risk of being viewed as naïve and uncomfortable when raising their voice. Some participants described situations in which they felt that they themselves or the organization were not true to their values, and they therefore chose to state that they did not agree with the decision made by the organization and discontinued their mission as a way to resolve the situation.

FGD 3: We were able to help many, so then you have to see it as that at least, that we could still help some and then, it was so that we, we tried to do most things, even if we were too few really to do all that, but it’s better to work a lot when we were there and try, as little as possible, to get angry. To get angry every day in trying to get others to do things they do not want to, then it is almost better to work more and then ignore, in becoming, having conflicts with the staff as well. So, it felt like that, so we decided we would do that anyway.

FGD 1: But there…we did not have a lot (of resources), like morphine you could not bring that in…//into the country and things like that so, we often had a lack of…and we were out of sterile gauze. Yes, but then we had to cut and sterilize ourselves and boiled over fire and all sorts of things because it was really hard with the logistics to get in (medical materials)…

FGD 1: That is ... a conflict that I recognize also, that it (is) as well as two sides and one wants to work ... impartial.// … It was mostly a rebel-controlled area… and we would work impartially but ... then we thought we got too few patients from the actual (x) ... area ... I noticed this more and more that it didn’t fit into (the organization’s) ... rules ... that you just take someone from one side, so this became a ... great conflict for me actually ... so I chose to cancel my mission ...

#### Consequences of unresolved situations: moral distress

This category comprises 14 subcategories that represent the consequences of not being able to resolve difficult situations.

Participants reported that the consequences depended on the severity of the situation, whether the situation was repeated, and the duration of the response. Several participants expressed realizing unforeseen consequences as an additional frustration. Feelings of helplessness, a surreal feeling, feelings of isolation and rumination were reactions reported as consequences by all participants. Several participants described feeling increased helplessness and isolation if it was impossible to resolve the situation or if support was lacking. Many participants noted that difficult situations stayed in their memory for a long time after the response. They were still thinking at the time of the interview of what happened and expressed that it had left them scarred. Some participants described that the difficult experiences “hit” them a long time after being home. They tried to forget and continue their normal work and daily life, but at some point, the experiences still affected them.

Some participants reported feeling exhaustion that led them to discontinue the response prematurely, while some reported that they discontinued in order to spare themselves before becoming too exhausted. Depending on their time in the field and their experience during the response, participants felt that their perceptions of coming home were affected in different ways. Some participants experienced coming home as more problematic than being in the field: some described that their feelings became obvious only after some time had passed and they allowed themselves to reflect. Some participants found that they had to adjust and find a new normal in their everyday life. Feeling alienated after repatriation was common among many participants, both at work and privately. Some participants described taking a break before they returned to their normal work. Another common reflection among participants concerned feelings of loneliness after the response, when no one was interested in listening to their stories, at either a professional or private level or both. Some participants described having lost a sense of naivety due to their experiences.

FGD 1: //...I think I was pretty ... I was pretty exhausted, or I needed to get home and somehow digest everything. And then ... I felt the risk was that if I were to stay ... then I would be burned out, and then maybe I wouldn’t want to go again ... while if I then stopped when I was still feeling okay... so ... I haven’t regretted that ... decision…

FGD 4: …This UFO feeling when you get home… and encounter Swedish healthcare. It… is there always… the longer the mission, the longer you feel like a UFO… And what people are talking about and what… the patients are talking about… You come from another planet, what is this? … But then it goes pretty quick… it goes faster for every mission, and the shorter the mission is… it is easier to just fall back into the… rhythm here at home…in some way.

FGD 3: It was really so difficult. And you, like, lose some kind of naivety. Or, and somehow, I feel that this ... // Yes, the world wasn’t as good. Or I do not know how to say ... But somehow ... // You lose something in that. Something you believed in or what it was now. And that, maybe you should have been warned about that before, that ... it won’t be so good afterwards, even if I don’t regret that I did it either, but maybe you would have been warned of how hard it was...

#### Consequences of managing difficult situations: positive consequences

This category includes six subcategories that describe experiences of resolving and/or managing difficult situations.

Many participants described the results of managing difficult situations as opportunities for personal and professional growth. Some participants stated that they could contribute to improvements due to their learned experiences; indeed, some expressed pride that their creative solutions and functional teamwork contributed to resolving issues. Several participants described that, in the end, managing difficult situations and learning from mistakes resulted in making a difference and achieving good for others. All participants claimed that when arriving home, they reflected on their experiences and learned things about themselves, which in turn gave them self-confidence.

All participants stated that they experienced positive consequences, such as a changed world view with increased awareness of inequalities among all participants, where they could be a spokesperson. The newfound awareness of inequalities was sometimes linked to a sense of guilt and sometimes to a sense of increasing gratitude. Many participants described, when returning to their work, increased reflection on the capacity of Swedish healthcare.

FGD 1: ... Or that it was like this, but you whine about the delayed bus or something like that ... // ... So, I think that somewhere, something happened in me as well or that I maybe got a slightly different outlook on the way in which I learned a lot when I was there but…

FGD 3: Mostly, you are very grateful to live here as a woman really. When you are exposed and when you see many other countries and how much you can decide over your own life, that is probably what you are most grateful for, I think when I return ...

FGD 3: … //…I have to say that I am grateful every time I put on gloves. I try not to forget that we have so much (of) here. We have plastic aprons and gloves and disinfectants all the time and can wash our hands.

### Theme 3, tools and support

This theme comprises four categories concerning the support and tools available to manage morally challenging situations and other ways to find support when it was lacking.

#### Informal support

This category represents five subcategories that describe access to informal support during and after the response.

Many participants described that it was mostly their own responsibility to take care of themselves during the response and upon their return home. When neither tools nor functional support were available to them, they had to find constructive solutions and support. Support during the response was described by almost all participants as coming from colleagues, with whom informal talks and relationships were seen as invaluable. Participants further reported that informal support was centred on having meetups in the evening or sharing everyday thoughts with a roommate. Several participants mentioned that it was invaluable to have someone who could understand and make them feel that they were not alone with their emotions. However, many participants described that this was dependent on the context, and if there was no one they could talk to, they would feel even more isolated. Another taxing circumstance described by many participants was the awareness of being unable to use their normal support network.

After the response, many participants described that it was difficult to share their experiences with family and friends. Three participants described that it would be unthinkable to talk about particular experiences with their partners, family members and friends. They therefore kept parts of their experiences and feelings to themselves. Several participants described that they chose to share their stories with a selected group of people whom they trusted or with persons who had undergone similar experiences. These persons could be individuals whom the participants had met in preparatory courses within their organizations. Several participants reported feeling relieved when they talked about their experiences with other people, realizing that they were not alone in their feelings of frustration and powerlessness during the response.

FGD 2: I did not tell everything (to my family)... not into the smallest detail ... because some things can be too extreme. //...Yes, just with security means and such things ... so like when you are in war and such things ... then it can be extreme around you, so ... like ... so everything in the smallest little detail, I don’t tell.

FGD 2: So, I'll probably say that, or to my rescue...// it was my local colleagues, that is, those who saw exactly the same thing as me. So, they were in it, and they were closer to it than I. They lived in the village, it was their neighbours, it was theirs...

FGD 4: You had so that ... then in the evening when you kind of switched off the lamp and would go to sleep then ... you could ... // ... because we shared rooms and came close to each other .... That was when you had ... that opportunity as well.

FGD 3: … And it becomes also, you also get debriefing during long time and, so that I think have been very important like keeping in touch with them (colleagues), because you have experienced a little, kind of similar things, yes. And it's often easier to talk through (when coming home), with those who have worked with similar organizations and so ... //

#### Formal support

This category represents three subcategories that describe access to formal support during and after the response.

Many participants claimed that appropriate and available formal support and tools during the mission were lacking. Leadership support within the team that was not functioning well added to the sense of frustration and loneliness. Participants contended that tools for ethical analysis were not used or that to their knowledge such tools did not exist, in their organizations.

Tools available during the response were described by several participants as being dependent on an individual’s own resourcefulness. They took their own initiative to find support during difficult situations; however, being provided with contact information for psychological support was described by two participants as giving them a sense of safety. Psychological support was used by only one participant, and this kind of support during the response was not provided by all three organizations. Some participants were able to meet with a psychologist at the expense of the organization after repatriation. One organization had a counsellor available when needed, whereas two organizations offered sessions with a psychologist after repatriation. Additional sessions could then be provided by a referral for psychological services within the regular health-care services. However, this was described by some participants as inappropriate due to the psychologist not seeming to understand what they had been through, or having no experience from the field. When the psychologist was unable to relate to the difficulties that participants had faced, this resulted in increased feelings of loneliness. However, they described it as helpful when a psychologist could relate to and confront participants’ stories about their experiences. After the response, debriefing sessions were mandatory within two organizations, and home-coming seminars were opportunities for reflections and sharing experiences. Home-coming seminars, which were provided by two organizations, were described as useful but difficult to attend because they were offered only a few times each year.

FGD 2: P2. ... I really think, I am completely pro all kinds of psychologists, but I still think that it is a triumph to meet other (colleagues) who have been in the same situation or in similar or completely different situations (to talk to)...P1: Like, you two ..? P2: Yeah exactly, it would have been great to meet some of you ...

FGD3: In (organization x) they have homecoming seminars, and so, then you can talk to others who have been in different places; you meet, and it is great because you ... it is like, here that you exchange, like, experiences from different places and then relate to it. A bit like that, so I still think that. Talking about it makes, after all, makes you see that it is not just you who has felt this way. Well, it’s more that thing that... helps in different situations so...

#### Preparatory support

This category represents three subcategories that describe the support given by the organization before the actual response which was helpful when facing difficult situations.

Preparation such as courses and trainings were described by all participants as useful to both their professional development and social networking. Participants argued that the social network developed from the courses was especially important. The network comprised a contact group with whom some participants had regular contact during their missions despite being in different places around the world. Briefings provided by the organization before the response were perceived by all participants as necessary but also sometimes as inefficiently designed or structured. Further, some participants reported frustration stemming from a lack of, or an ineffective, hand-over of information when arriving at the response site.

FGD 2: …we had a lot of group work and then, like one is supposed to struggle and do group work with someone who lives in another country and this, and everyone had different ideas about everything and everyone had different experiences. But after that, then we had each other. And then we have met a lot.

FGD 2: It was my first assignment. So ... you try to ... read in somewhat. But then it was not really what was in this description that I actually was doing at the time. Then, what I needed to know it didn’t say. Thus, such concrete details. If I’m going to write a referral on someone, how do I do it? Where should I make copies? Who will sign? Very handy stuff.

FGD 3: Neah, I think I looked them up (the hand-over reports) myself somewhere. No, that has been extremely bad. Extremely bad with those parts. These project-specific hand-overs…

#### Other tools

This category represents three subcategories that describe other tools and idiosyncratic methods used to manage and resolve difficult situations during and after the response.

Participants who used stress-management tools such as yoga or other group activities described these activities as meaningful tools to relieve stress. Further, group activities provided opportunities to get to know each other in the team.

Some participants described that they started to work immediately after coming home; however, this was sometimes experienced as very demanding. A few participants took some time off before returning to their regular workplace even though the hiatus was not arranged for by the organization. Several participants reported that if they started to work too early after repatriation, they became more frustrated in their regular work. However, some participants noted that professional colleagues were a source for support. Two participants described having colleagues who knew them well and who could recognize that they did not feel well and dared to ask about their wellness. Certain participants gave lectures and presentations about their experiences in the field when coming home. This was seen as a meaningful way to process their experiences. Participants who joined “post-project groups” with individuals who were participating in a specific response such as the Ebola outbreak considered the activity to be very useful: it provided an overview and understanding of what happened during the time, and participants could understand their own place in a larger whole. Several participants argued about the importance of keeping in contact with colleagues who had similar experiences, especially when, or if, there was little or no formal support.

FGD 2: It was: -Now, we will talk about all the investigations we’re going to do and //, so. And I worked on it, and then finally came, one afternoon the janitor came in (saying) like this: How are things really with you? How are you? Then it was the janitor, not a close colleague. – You’re not quite normal, are you all right? Here, it was so nice when someone just said that ...

FGD 1: What has been good for me, it has been to ... lecture. // Like in networks and like ... interest organizations and ... and schools and so. // ... So that ... it helped me through that in some way ... I think.

### Theme 4, engagement as a protective factor

This theme consists of four categories that cover participants’ perceptions of their professional role and motivation within their occupation which over all served as a protective factor despite the difficult situations they faced.

#### Altruistic motivation

This category represents two subcategories that describe an altruistic motivation to participate in disaster response. This motivation was stated by several participants to be the desire to achieve good for others, inform their community about the reality in the world, combined with an urge to make a difference.

FGD 2: ... what really ... happens because there were so many television pictures and ... that ... there was so much inaccuracy (through media) I also thought ... about these things, that it was ...then I felt a bit more that I //it was a bit my duty to tell as well, about these fates and a little about how it really is //...

FGD 1: But then there is something that you can think that it is exciting and that you think it is ... is fun but somewhere you will still do your best or do what you think or somehow,that is what makes the difference.

#### Professional and personal motivation

This category represents two subcategories that describe the professional and personal motivation to participate in disaster response and reflections on this type of work.

Many participants stated that their motivation to engage in this type of work was to get learning and experience. All participants suggested that their experiences gave them opportunities for growth. Gaining experiences as opportunities for growth outweighed the negative impact of difficult situations. Several participants described trying to remind themselves that regardless of the outcome, they had done the best they could with what they had. This was considered by many participants to be a way to cope and live with their experiences. However, this growth and confidence was something that they learned themselves and increased with the experiences from the assignments.

FGD 1: Seems to be like ... almost all people who go away ... That there is more than one assignment ... So, it points towards that ... there is something ... in it for ... // something in it for personal development.

FGD 3: But I can agree with that, despite being very unprepared, but I also felt that I was very ready with: ‘You can do this’. It is not your fault that things are not in place (when working in a disaster setting), that no one has checked, really, it is not your fault. You do what you can, and… And that I think you get with age, like that security…

#### Framed by the professional role

This category represents two subcategories that describe participants’ reasoning about their professional role within an organization.

Many participants described their awareness of their role within a larger structure: within this structure, they must follow rules and guidelines. This awareness resulted in the participants arguing that individuals cannot do what they choose at any moment. Several participants argued that the rules and guidelines are important and are there to protect the patients. However, the guidelines also serve to protect the local staff, since they should not be exposed to changing working conditions every time a new team member joins the response.

FGD 3. After all, it has to do with structures, cultures, more than the care itself. Because I buy that… the first paper I signed was that I would follow the organization’s guidelines and… I have accepted that. That I can’t do what I do here at home, there. But, on the other hand, one can have a humanistic attitude about what one does. That it is not a yes or a no, but one can always give good care and see that this is a human being, like...

FGD 1: According to the principles as well ... guidelines and ... there are often guidelines ... // if you talk about surgery or what you are talking about, antibiotics or whatever it is. // ... So that ... all employees then follow those. // ... That there is some structure with leadership and such, which gives some kind of control.

#### Challenges are part of the work

This category represents three subcategories that relate to the reasoning about facing difficulties and challenges as part of one’s occupation.

When discussing the overall experiences, participants expressed a common perception that there are problems everywhere and that the world is not perfect. Many participants related to challenges within their usual work in Swedish healthcare and perceived challenges to be a part of daily life. Another way of thinking reported by some participants was that they had agreed on the conditions of this kind of work and that this made them prepared to face difficulties.

FGD3: Yes, it is dilemmas, hospital beds and everyone who quits and how people leave for staffing agencies, and that costs us a lot of money instead of investing in those who are there and invest in nurses, in a just and decent working environment. It is horrible, it is really obnoxious when you work such shifts, I cannot cope with that ...

FGD 3: It’s like you go out and accept the medical ... part, that I cannot save a premature baby in (country x) even though she breathes, looks rosy and is alert. But I can give dignified care, worthy care.

### Theme 5, the work environment as a risk factor

This theme consists of two categories that cover participants’ perception of other sources of stress within their work environment. These stressors put additional strain to the participants when facing specific difficult situations which becomes a risk factor when dealing with moral stress.

#### Work environment

This category represents three subcategories describing risk taking and traumatic events in participants’ work.

All participants mentioned that they were aware of taking both safety and financial risks when signing up for this type of work. Being willing to take these risks was discussed as a factor that separated them from those who did not continue with this work. Some participants reported that they also took a personal health risk, since they understood that their experiences could affect their daily functioning when returning home. Some participants reported experiencing traumatic events, such as robbery and shootings, as additional sources of stress. These events, however, led to increased attention from the existing support structures: Participants reported that special support structures with evacuation possibilities and psychosocial support were quickly in place for those who had experienced such an event.

FGD 2: But that is a frustration that is quite common. In our assignments, people are hindered from travel because it is so difficult… so special ... those who go to Syria now and away to Iraq ... // So maybe you end up outside (the country) and are in some hotel ... at some strange place nearby ... and people tend to feel quite bad about that…

FGD 2: P1. It can be beautiful. When I see the pictures now, I see it, but didn’t I see how beautiful it was? But I don’t think I did. Because it was so difficult. P2: It was so, it was really tough. But it was because when I was there, it was so unsettled too, so you felt that ... everywhere it was turbulent, and you dared not go outside ...

FGD 2: Nah, but, but I think it was so much more concrete. // I mean a robbery and weapon, this all can be understood, and then they certainly have some PM then, because it has certainly happened, eh... But like this other, with which you go in this, uh, this reality every single day…

#### Stress and exhaustion

This category represents three subcategories describing additional sources of stress within their work.

All participants described facing difficulties within the team, especially when colleagues and individuals in leadership positions did not fulfil their managerial duties, as a factor leading them to feel drained. This resulted in further difficulties in obtaining support or raising issues within the team.

Many participants described their occupational context with its constant overwhelming needs and human suffering as being draining. This was especially a challenge as they lived within the context and did not have a chance to take a break from their working environment. Some participants also described having an issue with being in a role with responsibility within this context, in which they were supposed to function as a responsible team leader for their team.

FGD 4: … That’s what has been the most difficult… it was so difficult there and then, because there was no time to… reflect… //… like… we had… it was an emergency assignment where you were out… on the boat like fifteen hours a day and then go home and prepare for the next day… // and then up and go to the next and… there was someone… new as well… similar story that you met… so it was like…//… It was not so easy to handle it there and then you only got to … // ... push forward

FGD 2: Pregnant women ... with small children and rubber boats and ... only that, you get so influenced by how miserable...how horrible people have it and that they still have the power to ... take this journey ... So yeah … it is also that you are weakened by...just by the situation ... Emotionally within the situation, in many situations…//...you see so much suffering...which might weaken you...already before a specific event happens… I can think …

## Discussion

This study explored how disaster healthcare responders perceive and manage moral challenges and how they perceive these moral challenges in relation to their wellbeing. The results indicate that disaster healthcare responders perceive morally challenging situations related to the profession as inevitable within their work. The participants described that the effects of these moral challenges lead to both negative and positive effects on wellbeing. Further, the findings suggest that if there are internal and external resources and capacities to manage and resolve situations, this can lead to positive consequences such as building confidence, growth and learning. However, if the situation cannot be resolved and capacities and resources are lacking, this might lead to moral distress. Participants also reported that they had to find their own support, as they often perceived a lack of functional formal support during their response. Useful support after repatriation included being able to discuss their experiences with people who could relate to the disaster response context, whether it was with a psychologist, a peer or in group-seminars. Further, the responders perceived that moral challenges had negative effects on their wellbeing depending on the severity of the situation, whether it had a repetitive nature and at the same time facing additional sources of stress and exhaustion in the work environment.

### Moral stress perceived as inevitable and real

The findings in this study indicate that moral stress and moral distress are experienced by responders and that they affect participants in different ways. Morally challenging situations were not described as something new; participants reported that they encountered similar issues related to medical decisions within their day-to-day work at home. Nevertheless, when working as responders, they faced a broader range of moral challenges, as such a context entails extraordinary situations deriving from limited resources and greater needs. However, morally challenging situations arising from colleagues’ wrongdoings or differing motivation were stated as a surprise and something they were not prepared for.

### Self-taught to deal with moral stress and moral distress

Participants used their own resourcefulness and creativity to deal with moral challenges and moral stress. They taught themselves to deal with difficult situations and reported that they developed experience and wisdom from them. The experiences resulted in a changed worldview; however, this was something they had developed themselves and came with a cost: lost naivety. Further, they argued that they had learned to reason that they had done their best with the means they had, perceiving that they had to play a role in an unperfect world. Therefore, they learned that the circumstances were not their own responsibility.

### Positive consequences of moral stress and moral distress

Further, the study results pinpoint that moral stress and moral distress can lead to positive consequences, not only negative consequences. Participants argued that they learned more and more after each response and that their experiences led to personal and professional growth. When they continued to try to do their best, it meant that they could somehow contribute and make a difference; the positive consequences outweighed the experiences of dealing with difficult situations. However, the findings may suffer from survivorship bias, because the study participants were those who still work as responders and are still engaged within their organizations. Therefore, we cannot make any statements about those who chose to leave their organizations or their professions due to the consequences of moral distress or moral residue.

### Similarities and differences with respect to moral (di)stress among healthcare workers during normal healthcare provision

In a study among Canadian paediatric nurses, similar types of morally challenging situations were highlighted as contributing to moral distress: perceived futility of care, unethical behaviour, poor communication, powerlessness, burdensome technology, delayed end-of-life care, provision of false hope and unnecessary testing [5]. Most of these situations correspond to our study results regarding morally challenging situations among responders, except for burdensome technology and delayed end-of-life care. Another study from Belgium, a quantitative literature review of moral distress among nurses, highlighted similar situations: a negative ethical climate and/or futile care, which contributed to the intensity and frequency of moral distress. These situations were mentioned by these authors as the most common moral challenges appearing within most cultural contexts, although within the normal health care context and not a disaster setting [16].

The same study from Belgium found incongruence regarding reported perceived moral distress and age among healthcare workers [16]. The authors argued that different cultural and context-specific factors could play a role in how nurses learn how to deal with ethical challenges. Older nurses may have more effectively learned to cope with ethical challenges. On the other hand, the accumulation of experiencing moral distress increases with the number of years in the profession. Therefore, they argued that some older nurses may be accustomed to moral distress due to experience, while others may be troubled with cumulative moral distress or moral residue [16]. In another review study, the author argued in parallel regarding the intensity of moral distress, noting that senior nurses might be less affected by moral distress as they gradually become accustomed to it while also being more vulnerable to increased moral residue. Additionally, those with longer experience are exposed to increased frequency since they face moral distress more often [36]. This links to our findings about responders’ moral distress and experience, even though the participants did not discuss their age as a major factor. However, the participants stated that they gained wisdom and growth through their experiences, which they did not possess before their first assignment. There is a double-edged sword here; with experience, the responders learn how to deal with moral challenges, but at the same time, they are exposed to increased consequences of moral distress since they face exceptional moral challenges to a greater extent within the disaster context than in normal healthcare settings. Longer experience is not necessarily equivalent to increased frequency of moral distress per se, as it depends on the level/grade of moral stress, the individual’s capacity, the level of support and other factors within the context.

Further, the professional hierarchy and social power within the profession are mentioned as influencing the intensity of moral distress [36]. The results of our study show that the disaster context does not make responders less prone to issues related to social power and professional hierarchy even though the context itself would involve increased team collaboration. This was also mentioned by authors in two studies among ICU clinicians in Belgium and USA, where interdisciplinary collaboration and positive institutional culture created a good ethical decision-making climate among team members and could therefore decrease moral distress [13, 14].

Strategies for resolving moral distress were reported in the study of nurses from Canada, mirroring the strategies that the responders in our study used [5]. The strategies were described as active, reflective and external; the active strategy relates to one’s own personal actions to address moral distress, the reflective strategy is related to perspective taking, and the external strategy is related to creating and identifying formal support. Further, the authors argue that experiencing moral distress cannot be eliminated since the interplay of differing factors is a part of the work context; instead, interventions should focus on developing personal growth and moral resilience. The responders in our study reasoned in the same way: they perceived difficult situations as inevitable and tried to deal with the consequences of those to the best of their ability. They were self-taught in developing moral resilience; however, they did not have much of a choice, as they lacked functional support and specific preparatory training in dealing with moral stress. This relates to the findings from a study about Chinese disaster healthcare rescuers’ resilience, where adequate preparedness and training appeared as important components for developing resilience in rescuers to deal with difficult situations [12].

### Refined conceptual model of moral stress and moral distress

**Fig. 1:**
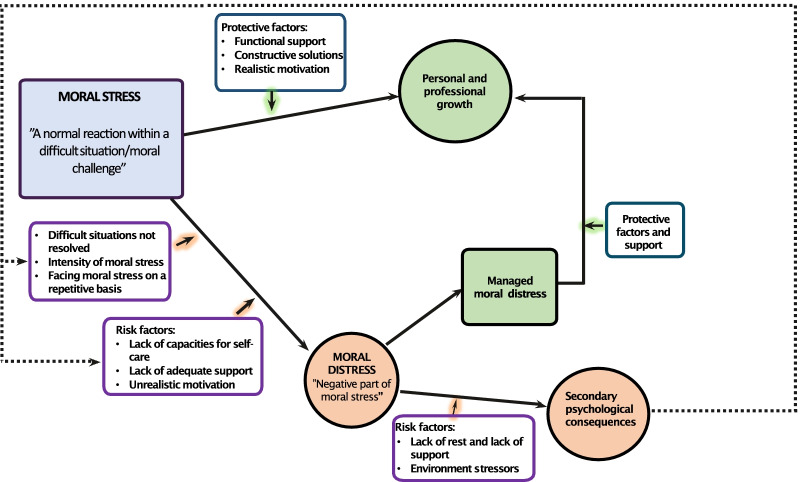
Conceptual model of the development of moral distress and its consequences

The findings in this study relevant to the development of risk- and protective factors of moral distress will be compared to the different steps in a developed conceptual model of the development of moral distress by Gustavsson et al. [3]. The findings in this study provide increased knowledge of the risk- and protective factors regarding the development of moral distress. A refined conceptual model was therefore developed to illustrate the development of moral distress in this study (Fig. 1).

A difficult situation highlights a moral challenge that produces feelings of frustration, powerlessness, helplessness; moral stress. Those who were able to resolve a morally challenging situation experienced positive consequences. Such positive consequences are added to the conceptual model with an arrow pointing to a new box labelled *personal and professional growth.* However, if there was no possibility of resolving the situation, participants described feelings of moral distress; powerlessness, rumination, helplessness, frustration, and isolation. Healthcare responders relied on their own capacity to manage and resolve moral stress, where a balanced motivation seemed to be protective against moral distress. The length of the response and having opportunities for recuperation during or between their shifts and their missions also seemed to affect their capacity to manage morally challenging situations, these are labelled as *protective factors* and added to the conceptual model. The work environment included potential security risks and being prone to financial and personal health risks, created additional stress factors (see *risk factors* added to the conceptual model). Professional responsibilities had to be addressed in an environment with constant suffering and sometimes a malfunctioning team, which made participants prone to exhaustion and resulted in fewer resources for them to manage moral stress and moral distress (see *risk factors* added to the conceptual model). Further, for those who were able to manage moral distress depending on the availability of support and other protective factors (see *support and protective factors* in the model), a new box is added to the conceptual model labelled *managed moral distress*. It is difficult to fully elucidate the difference between moral distress, moral residue and other consequences, such as burnout among the participants in this study. However, moral residue seems to be the ruminating and lingering feelings regarding moral challenges during a longer time period. Still, the participants in this study mentioned personal and professional growth despite their perceived moral distress which could point at not experiencing secondary consequences to a larger extent. However, risk factors might influence the level of these secondary consequences, and exhausted responders could then have correspondingly fewer resources to deal with future moral challenges. However, more research is needed to elucidate how moral distress are related to secondary psychological consequences. Therefore, in the conceptual model, an outside dashed arrow is added to represent that those secondary consequences might negatively affect the capacity to manage future moral challenges and moral stress.

The study results can be useful for organizations involved in disaster response, to inform how responders perceive and manage moral challenges and which support mechanisms that would be beneficial, before, during and after assignment. Further, the results might be useful to include in preparatory courses for disaster health care responders. Moreover, some of the situations that participants described in this study are similar to those that health care personnel encounter in the current COVID-19 pandemic in Sweden and other countries; therefore, the findings can be useful to consider in health care organizations’ support structures.

### Methodological considerations and limitations

In this study, the descriptions and experiences of difficult situations and its effects (moral stress and moral distress) are reported by the participants themselves. Therefore, it is their view of what kind of situations that was perceived as moral challenges, how these were managed and how it affected them.

Further, since this is a qualitative study with a small number of participants, we cannot know whether the reactions of the study participants can be generalized to responders as a whole. We can only mirror the views and experiences of the responders in this study who are still engaged within their organizations. Further research is needed among responders who have left their organization, perhaps due to consequences of moral distress, to better understand the various effects on wellbeing.

The study participants had different roles with various levels of proximity to patients and were deployed from Sweden through three different organizations to different disaster contexts, which might have affected their intensity of moral stress and perception of difficult situations. Further, the participants had differing authority within their professional role, as nurse or physician. However, the results indicate that their experiences of moral challenges were more similar within disaster settings than working within normal health care settings. Hence, the two professions’ experiences of moral stress were not described separately. Nonetheless, it may be fruitful for further research to investigate differences related to professional roles. Further, we do not know if health care workers from high-income healthcare contexts with a high motivation to assist in settings with lower resources could experience higher grades of perceived moral stress. More research is needed on how local disaster responders in low resource settings perceive moral challenges within their own context. However, the aim of this study was to investigate how responders perceive and manage moral challenges in all types of responses to provide a broader understanding of various moral challenges.

The results in this study represent participants’ views and experiences and therefore provide unique insight. The study results display some similarities with experiences encountered by health care workers in high-income settings. However, further research is needed within the disaster context to also explore the differences and similarities related to experiences among local responders and international responders. In addition, further research is needed to assess and quantify perceived moral stress and moral distress within a larger group of responders.

## Conclusions

In this study we explored how deployed disaster healthcare responders manage and perceive and are affected by moral challenges. The participating disaster healthcare responders were self-taught to deal with both moral challenges and moral distress. Moral challenges were described as inevitable and predominant when working in disaster settings: such challenges had to be dealt with. The type and length of the response and also the severity, the frequency and the duration of the morally challenging situation was perceived as negatively affecting the wellbeing of the responders. Support considered useful was foremost collegial support, while psychosocial support after deployment was considered useful provided that this person had knowledge of the working conditions and/or similar experiences. Further, being informed of and prepared for facing differing moral challenges in a challenging work environment could be helpful to manage moral stress. The difficult experiences might also lead to positive effects such as personal and professional growth and a changed worldview, although at a personal cost. This study provides unique insight into disaster responders’ perceptions of moral challenges in relation to their wellbeing, which could be integrated into organizations’ support structures for responders before, during and after deployment.

## Supplementary Information

**Additional file 1: Appendix 1.** Interview guide.

**Additional file 2: Appendix 2.** Chosen excerpts.

## Data Availability

Relevant data will be available upon reasonable request from the authors.

## Abbreviations

FGD: Focus group discussion
SRC: Swedish Red Cross
MSF: Medécins sans Frontières
MSB: Myndigheten för Samhällsskydd och Beredskap/Swedish Civil Contingencies Agency
