# Supplementary material for: Dealing with difficult choices: a qualitative study of experiences and consequences of moral challenges among disaster healthcare responders
Source: Confl Health. 2022 May 8;16:24. doi: 10.1186/s13031-022-00456-y (PMC9079207; doi:10.1186/s13031-022-00456-y)
Supplement: Supplementary file 1 — Additional file 1: Appendix 1. Interview guide. [file 13031_2022_456_MOESM1_ESM.pdf]

S2. Appendix 1. Interview guide

Interview guide

Introduction:

You are all here today due to that you have unique experiences from working in emergency- and disaster response within different organizations. Here you meet other individuals with similar experiences to collectively discuss and share opinions within this specific subject. The discussion will be recorded, and the data will be treated confidential, things that are said within this room stays here, just in line with the information you have received beforehand. When the results are compiled you will receive the report, and your organizations as well. The result will not be linked to you personally, and the organizations cannot know who has accepted to participate in these groups, or not. If you have more specific questions regarding the aim of the study, we can have a chat after the group discussion.

The aim of a focus group discussion is to reach a collective understanding/experience within a certain subject. So, this will not be a "debriefing session", instead a focused discussion around your collective experiences. There are no right or wrong answers, all thoughts and comments are important, so please share your opinions with us. We might interrupt or guide the discussion so it is centered and focused on the subject, otherwise you are welcome to add and fill in into your colleague's comments which will contribute to nuances and variation. Our role (as moderator and assistant) is to keep the discussion focused around the subject and to encourage everyone to contribute and share your angles of incidence, in a relaxed way.

Interview guide:

Working in disaster and emergency response involves various challenges, ranging from lack of resources and time combined with overwhelming needs, and a chaotic environment. This often mean that you are exposed to difficult situations where each choice of action might feel wrong, or that you feel that you cannot act upon what you perceive is right according to your values, due to external constrains. This may result in that it "feels difficult". (Feelings of frustration, powerlessness, inadequacy, regret, etc.).

Let us start with that you tell us about a situation which felt "difficult", after that we will have a more general discussion about consequences, tools to deal with the situation, and how one might be affected -on a professional and personal level.

1. Could you tell us about such challenges (with difficult choices or when you could not act upon what you felt was right) related to your professional role during your mission?
   Choose one situation that you would like to share. Keep in mind that everyone need time to talk.
2. What did the situations (the choices) center around?
   Was it the decisions that felt difficult or was there something else in the situations that felt difficult/challenging? Eg. patient-related treatment, priorities, unpredictable consequences, cultural circumstances, organizational structures, acting outside of competence, team-collaboration, be associated with/affected by others' decisions/misbehavior etc.
3. What was the consequences for you by this difficult situation? How do one feel when confronted with these situations?

(What did the decision lead to? What did your actions lead to?) What might be the consequences of these feeling/what can these feelings lead to?

4. How is one's workability affected by these difficult situations/decisions?
   On a personal level and on a professional level. How was your professional role affected? Could you continue working? There and then?
5. Was there something that made the situation easier during/directly after the situation?
   Was an analysis or survey of the situation made in the field/mission? Structured debriefing in the field/mission? Does the organization have guidelines for this?
6. Was there something that made the situation worse -right there?
7. Did you have any tools that was helpful related to your decision-making?
   Eg. medical guidelines, ethical guidelines, discussions with colleagues, support from leadership, earlier experiences. How were you met if/when you spoke out/asked for support?
8. When coming home, how can these difficult situations affect individuals (you)? (How have you been affected by the decisions/priorities that you made when coming home?)
   Do you still think of your decisions today? When you came home? And at your work at home? Work-life vs private life?
9. With your knowledge today, do you have any advice or recommendations about how individuals could deal with these situations with these challenges?
   Could you have been prepared for this? Is it possible to prepare? What could be the role of the organization, before, during and after? Before you left to the field where you prepared for getting yourself into difficult moral situations?

Do you have anything else that you want to add? As you notice, you are not the only one with these feelings and your experiences you have discussed here could be useful to others. If you have questions that pop up, or something you think of after, please feel free to contact us. Thank you for sharing your stories and experiences.
