# Supplementary material for: Dealing with difficult choices: a qualitative study of experiences and consequences of moral challenges among disaster healthcare responders
Source: Confl Health. 2022 May 8;16:24. doi: 10.1186/s13031-022-00456-y (PMC9079207; doi:10.1186/s13031-022-00456-y)
Supplement: Supplementary file 2 — Additional file 2: Appendix 2. Chosen excerpts. [file 13031_2022_456_MOESM2_ESM.pdf]

S3. Appendix 2. Chosen excerpts

1 Theme 1: Type of difficult situation: duration, frequency and intensity

1.1 Category: Not being able to act and being hindered by external circumstances 1.1.1 Subcategory: Politics

"FGD 2 ... We took care of, we were in (this conflict area). Then we were at a, (the organization) assisted a hospital there. An existing one, but then we would just take care of patients from (the other area). And it was very... hard for especially the, local, local employees they were, didn't want to take care of these at all. "

1.1.2 Subcategory: Difficult priorities

"FGD 1: Yes, as some kind of ... justification for these types of decisions was like ... which I thought you got ... We talked a lot about (it) and so .... the hard fact was that we were actually there more to fight an epidemic than to help individuals actually."

1.1.3 Subcategory: Cultural aspects in the work context

"FGD 3: I think that is what you are trained in as well, and that is what you are more used to handle. But these other things we are not, that is, at least not on my level that you are not organizationally trained and like sitting, I do not know, at meetings and so and write guidelines and everything like that. Or, I was not that at all so, it is there the frustration and when your own organization is working against you, who is there to help you, I have experienced it. But the patient-meeting is something that you have been trained in before..."

1.1.4 Subcategory: Lack of resources and enormous needs

"FGD 2: And it was full ... it was full war ... there were ... so very badly sick people and injured…multi-traumas...there were horrible orthopaedic things because they were, eh. … But ... It became so much so I didn't know what... I had to do something, I couldn't really do anything…or really should not. We should only evacuate; we should only be there."

1.1.5 Subcategory: Being hindered by the organization

"FGD 1: ... with the Ebola then it was two short (missions) ... then I felt one kind of frustration the first time ... because then I was like ... responsible doctor then so ... one had decided that it should be so short ... and there was high turnover of people ... that ... one like lost in tempo ... in like ... the institutional memory kind of disappeared …"

1.1.6 Subcategory: Not being able to act

"FGD 1: P1. Because we do not have any equipment, we have no diathermy, we did not have electricity…nothing //(it) is not possible to blood still and it was a pretty hopeless surgery, but then they started and performed one, and then there was no recovery ward, nothing, not even a bed to lie on.// (He) got lying intubated with some ventilator during the night and then (we) came back, but then he lived ... Yes, but then one did an additional surgery and then later he died ... this, I perceived ... as very unethical that they... that they (did this) …P2. It became like an experimental workshop everything."

1.1.7 Subcategory: Not being able to give care and comfort

"FGD 1: Yes and this thing to be there for patients who had anxiety and to stay with them, it was like ... (impossible) ...// And during the first round so ... then it was a boy who had anxiety ... and when we came back next time ... then he was dead.// That picture has been like stuck in my mind, it has ... Ehm "Yes, we will come back" like."

1.1.8 Subcategory: Own security hindering giving care

"FGD 1: We talked a lot and we had a midwife in our team who was a promoter, health promoter, so she took care of these women and went in and like ... Wanted to... stay with them. But we literally pulled her out of there. She became too emotionally engaged as a midwife with these women ... So, we had to ... // ... more or less force her to also accept the decision that had been taken ... "

S3. Additional file 2, Chosen excerpts

1.2 Category: Actions perceived as insufficient

1.2.1 Subcategory: Not standing up for values

"FGD 3: Because you put higher demands on those that you work with, of course. It is us, or like, we must, someone must ... stand up for what we do. And that I often think makes it more frustrating than the work in itself – if the organization does not support you in what you do or, yes. And not only, not just, like having opinions but also, sort of simply facilitate the work."

1.2.2 Subcategory: Making different priorities

"FGD 1: P2. But I was thinking that when I was there ... my role as a doctor and (to) do exactly the right treatments and things like that got a pretty serious setback so to say... "

1.2.3 Subcategory: Frustration in not having enough capacities to act sufficient

"FGD 1: …But you are still not prepared anyway, because of the heat and… and to manage like ... // So in a short time, you want to do so much more ... I was a supervisor nurse then ... had 60 patients ... in high risk and ... and had four tents."

1.2.4 Subcategory: Inequalities between you and the population/local colleagues

"FGD 3: Yeah, it was totally absurd, like ... Yes, so, I have felt so badly bad for this. Because it is like (our) state, it is our tax money. And we got food that was in top class, for me in restaurant top class, so we ate fillet of beef and then they had calculated 400 g per person of fillet, so we sat and ate at the "restaurant" and like burped garlic and came out to the refugees and then they got a half of white bread and a half apple. That's just one example. It was stuff like that all the time."

1.2.5 Subcategory: Balancing collaboration with other partners and authorities

"FGD 1: ... or he told me, it was pretty hard for me to hear then, but that ... That really, we were there and gave ... it was (like) a trading almost, that we offered this healthcare because then they agreed to negotiate other things as well and I remember that I thought it was quite like this... to realize that okay, it is not // ... the healthcare which is so important but it really is the thing that they agree to give concessions to other things if we offer this."

1.2.6 Subcategory: Having to build trust before acting

"FGD 3. ..Eh and then it was also a challenge that you must…work together with ... (the authorities) because they have not said that this was a disaster in that way, but everyone was, they asked for help but not in that sense that you can only come in and ... do what you want."

1.2.7 Subcategory: Frustration due to lack of coordination

"FGD 3: P1. There were very many organizations. That is why it gets so messy. P2. If no one takes the command as well..."

1.2.8 Subcategory: Frustration due to lack of leadership

"FGD 1: ... more and more (personal) conflicts ... and was like ... // more and more tired, as well as ... that it turned out to ... a bad ... atmosphere also due to that they prolonged with people which should not ... really ... // so, then you could not ... But they did not find any other, so because it would look good on paper in some way, so they like ... "No, but we need an anesthetist, so we prolong his (contract)"… //But it became like not (good) ..."

1.3 Category: Imposing harm instead of helping

1.3.1 Subcategory: Creating false hope

"FGD 2: It (the boat) was quite big anyway, hefty as well, and when this fantastic boat is revealed to people who are sitting in a rubber float, of course you think you should (be brought to a safe place)…"

1.3.2 Subcategory: Causing harm instead of helping

"FGD2: They had planned for this trip and they might have reached Europe with that boat and they probably had even been better off, because then they would have been able to eat their packed food and things which we took away from them ... And then it was a conflict with those who were in the other ... Well, this I think ... I wasn't really prepared to end up in a class society on the aft deck of the boat..."

S3. Additional file 2, Chosen excerpts

1.3.3 Subcategory: Different motivation and attitudes among colleagues causing bad reputation

"FGD 3: And then I experienced, huge ethical dilemmas, when I saw that those who worked there, not at all were humanitarian. It was really hard for me and I still have a hard time with this... When I work for (organization x) we gave food to all refugees, in a dose that is, a normal food-dose as well ... But there they would receive half an apple and a half sandwich, per day".

1.3.4 Subcategory: Being part of a bad decision or act

"FGD 3: P1. She didn't want to give these medicines that were not really right. In the end, you know, I would also feel like… if you yourself get prescriptions that you yourself do not think is okay, then ... How to, Yes, what should you do then? … If you do not give them, it feels like, then I do something that is not okay either, as well. P2. You are sort of included in that decision ..."

1.3.5 Subcategory: Colleagues wrongdoing

"FGD 3: And it was a nurse who worked with him who said that yes, unless he leaves, then I leave. And which she later did, she left because she couldn't work with him. She had written down every day what he had done: He told the patients this, he prescribed this antibiotic, he did this, and, like, she had documented ... for a month, wrong, wrong, wrong. But, in the end, she gave up, like no, this doesn't go, I can't."

1.3.6 Subcategory: Being accused for a decision taken

"FGD1: But we work in healthcare as well, we are used to that kind of people ... like you are unable to manage certain patients within surgical care also ... // ... like here at home ... // ... But it is this thing that you can stand there by yourself, like this decision, like, I ... the example I had. //...That you stand alone with the decision yourself and that you then, by a colleague, can be (accused) like this."

1.3.7 Subcategory: Contributing to or causing inequalities

"FGD 3: P1: …So, this cultural perspective, and that, this hidden or open racism. I think that is the worst thing really. We and them. P2: We or them. That is used often I think, I have encountered. // Both by expats, but also by national (staff) …"

2 Theme 2, Managing difficult situations

2.1 Category: Resolving the situation

2.1.1 Subcategory: Constructive solutions

"FGD 1: But there…we did not have a lot (of resources), like morphine you could not bring that in…//into the country and things like that so, we often had a lack of…and we were out of sterile gauze. Yes, but then we had to cut and sterilize ourselves and boiled over fire and all sorts of things because it was really hard with the logistics to get in (medical materials) …eh…"

2.1.2 Subcategory: Focus on solutions rather than problems to adopt to the context

"FGD 3: We were able to help many, so then you have to see it as that at least, that we could still help some and then, it was so that we, we tried to do most things, even if we were too few really to do all that, but it's better to work a lot when we were there and try, as little as possible, to get angry. To get angry every day in trying to get others to do things they do not want to, then it is almost better to work more and then ignore, in becoming, having conflicts with the staff as well. So, it felt like that, so we decided we would do that anyway."

2.1.3 Subcategory: Create collaborations and ask for help to resolve

"FGD 4: …Very difficult when you come from the outside. How much should I put in? How much can I put in? Not at all or ... can I still have some role ...? But then we managed to get some out of his ... // ... we talked to the family on the phone ... and some from his church came there ... his brothers ... and so we would discuss his funeral ..."

2.1.4 Subcategory: Using experience

"FGD 1: The second time I was there ... we had more experience and then ... we were less afraid, but then it was also at the end of the epidemic so not as many patients."

S3. Additional file 2, Chosen excerpts

2.1.5 Subcategory: Do something instead of nothing

”FGD 3:… I usually think of this that sometimes you do something only because you have to yourself as well. ... he surely died anyway, most likely, but ... right then, I felt like I was doing something ... the only thing I could do. Then of course I had a very hard time ... replacing ... (the drug) that we had ... in our bag that we would have for my colleagues ... in case of ... Yes, you can do stupid things like … which feels like the only solution ...”

2.1.6 Subcategory: Sense of teambuilding

"FGD 1: When we were inside and worked in this zone and so, it really did not matter -doctor or nurse or anything else, you just had to help out and do a lot."

2.1.7 Subcategory: Fighting and using courage to resolve

”FGD 3:…Which I thought was completely absurd, so I fought like crazy, until we got, at least succeeded in doubling the dose, so that in any case they got one apple and one sandwich a day at least.”

2.1.8 Subcategory: Discontinue the mission

“FGD 1: That is ... a conflict that I recognize also, that it (is) as well as two sides and one wants to work ... impartial.// … It was mostly a rebel-controlled area… and we would work impartially but ... then we thought we got too few patients from the actual (x) ... area ... I noticed this more and more that it didn't fit into (the organization's) ... rules ... that you just take someone from one side, so this became a ... great conflict for me actually ... so I chose to cancel my mission ...

2.2 Category: Consequences of unresolved situations: moral distress

2.2.1 Subcategory: Realizing unforeseen consequences

“FGD 4: And then what happened was that when the assisting doctor, he…//…told me continuously...how it went with…the family to him who died.//Because then they were put in quarantine by…their neighbors, they could not go outside…//…during three weeks.// So they did not have food and they did not have…So…Yes…then we tried to…solve that…instead, so there was a continuation in this…with this family.”

2.2.2 Subcategory: A sense of helplessness and ruminating

“FGD 4:…Because when you are just in the middle of it… then you have no idea… Or the first thing was that I really… is there something more… should I have made something more medical? What could I have done ...? Could I have, in some way, saved him?”

2.2.3 Subcategory: Feeling isolated and surreal

“FGD 3.… It only increased; it increased every month. And ... I felt very powerless and no matter what we did...so, only more and more in some way ...”

2.2.4 Subcategory: Feeling scarred

“FGD 2: And this still lives with me. This (situation) I can feel now that I am still reacting on it. And I have noticed that earlier also, that is why I thought about this one. // It was so much. But eh…it…it was a huge learning for myself. I know that I made it. I can handle quite a lot…”

2.2.5 Subcategory: Not worth it, leaving earlier

“FGD 2: Yes, I actually did that. I actually interrupted // ... I was, I was completely exhausted. I had nothing more to give. And there was no reason for me personally, I had nothing left to prove ...”

2.2.6 Subcategory: Leaving earlier, to save yourself

“FGD 1: //...I think I was pretty ... I was pretty exhausted, or I needed to get home and somehow digest everything. And then ... I felt the risk was that if I were to stay ... then I would be burned out, and then maybe I wouldn't want to go again ... while if I then stopped when I was still feeling okay... so ... I haven't regretted that ... decision…”

S3. Additional file 2, Chosen excerpts

2.2.7 Subcategory: Depending on the time in the field

”FGD 2 :. //…Then I went on a short (mission) and it was a completely different thing. //…only to know that you will soon leave from there, or also that it did not ... you do not dive into all these small... difficult things all the time, or these little things as well.”

2.2.8 Subcategory: More tough to come home than to be there

“FGD 2: Mhm, but since you say, the hardest thing for me was absolutely to come home. That was much harder than to be there.”

2.2.9 Subcategory: Finding a new everyday life

“FGD 4: So, then there it became a very long ... break ... even though I felt that it maybe came into an everyday life here at home so ... I couldn't ... either let it go ... the experience either ... It was like a parallel life during …quite a long time."

2.2.10 Subcategory: Feeling alienated

”FGD 4: …This UFO feeling when you get home… and encounter Swedish healthcare. It… is there always… the longer the mission, the longer you feel like a UFO… And what people are talking about and what… the patients are talking about… You come from another planet, what is this? … But then it goes pretty quick… it goes faster for every mission, and the shorter the mission is… it is easier to just fall back into the… rhythm here at home…in some way.”

2.2.11 Subcategory: Feeling lonely

“FGD 2: And then maybe you think that you are "Normal", eh, but someone who can put you against the wall maybe, and (make you) come down. I have had two (persons), when I came home from (x) and when I had been in this. Then I went straight back to work //. So, poof, and everything would be like (normal), and everyone first asked like this: How has it been? But then, nobody really wanted to know…”

2.2.12 Subcategory: It eventually hits you

"FGD 4: And I probably did not really understand ... for a long time either ... to what extent it was with me, because I ... // ... sort of came home and started ... studying and throwing myself into something quite different and ... as well continued to push this away. ”

2.2.13 Subcategory: Losing something

"FGD 3: It was really so difficult. And you, like, lose some kind of naivety. Or, and somehow, I feel that this ... // Yes, the world wasn't as good. Or I do not know how to say ... But somehow ... // You lose something in that. Something you believed in or what it was now. And that, maybe you should have been warned about that before, that ... it won't be so good afterwards, even if I don't regret that I did it either, but maybe you would have been warned of how hard it was... "

2.2.14 Subcategory: Having a pause before going back to work

”FGD 2: Yes, that's the best thing I've done in my whole life. I mean ... to not go back to work then. To just be at home... I was ... I was totally fatigued.”

2.3 Category: Positive consequences of managing difficult situations

2.3.1 Subcategory: Contribute to improvements and better support

"FGD 1: .... that you then ... through ... // ... some stories might, like ... // ... maybe ... influence someone, or like be able to tell how...a bit how it really was then.”

2.3.2 Subcategory: Creative solutions

"FGD 3: … I flew down with a water engineer and we didn't have the same, at all, view of things… Even though we should try to work together and try to reach (a solution) … but in the end it turns out well. You learn from each other anyway and learn, to think practically and so, as well. So, it's also interesting, you learn a lot in that way too.”

2.3.3 Subcategory: Learning from mistakes

"FGD 1: Yeah ... you should like, see this ... on a global... in a big perspective, then it was only two individuals ... two patients. //…and when we work like this, we have to think more in a public health perspective, like more in the context and try to ... for one's own ... rescue reason ... try to think that .... you do the best ...all the time. "

S3. Additional file 2, Chosen excerpts

2.3.4 Subcategory: Self-confidence and learning things about myself

"FGD 1: ... Or that it was like this, but you whine about the delayed bus or something like that ... // ... So, I think that somewhere, something happened in me as well or that I maybe got a slightly different outlook on the way in which I learned a lot when I was there but…"

2.3.5 Subcategory: More aware of inequalities

"FGD 3: Mostly, you are very grateful to live here as a woman really. When you are exposed and when you see many other countries and how much you can decide over your own life, that is probably what you are most grateful for, I think when I return ..."

2.3.6 Subcategory: Growth and being more grateful

"FGD 3: … Plus this abundance. But you quickly pass that and learn that we have. But on the other hand, I have to say that I am grateful every time I put on gloves. I try not to forget that we have so much here. We have plastic aprons and gloves and disinfectants all the time and can wash our hands."

3 Theme 3: Tools and support

3.1 Category: Informal support

3.1.1 Subcategory: Relying on informal support while there

"FGD2: So, I'll probably say that, or to my rescue...or as well as possible, it was my local colleagues, that is, those who saw exactly the same thing as me. So, they were in it, and they were closer to it than I. They lived in the village, it was their neighbors, it was theirs..."

3.1.2 Subcategory: Creative support-seeking while there

"FGD 4: You had so that ... then in the evening when you kind of switched off the lamp and would go to sleep then ... you could ... yes ... because we shared rooms and came close to each other .... That was when you had ... that opportunity as well."

3.1.3 Subcategory: Lack of access to normal social support while there

"FGD 3: Yes, I talked to others ... and when we had ... network I might be able to write to those who, know ... my life situation ... then you, sort of talked about it ... But that is a bit special when you are away... You do not have the natural, contact with your regular network. Partly due to that you do not have any internet...but that you do not have any usual people like that ... to brief with ..."

3.1.4 Subcategory: Lack of social support when home

"FGD 2: I did not tell everything... not into the smallest detail ... because some things can be too extreme. //...Yes, just with security means and such things ... so like when you are in war and such things ... then it can be extreme around you, so ... like ... so everything in the smallest little detail, I don't tell."

3.1.5 Subcategory: Creating your own support when home

"FGD 3: … And it becomes also, you also get debriefing during long time and, so that I think have been very important like keeping in touch with them, because you have experienced a little, kind of similar things, yes. And it's often easier to talk through, with those who have worked with similar organizations and so ... I think. After all, it is the same who lives here in Sweden also."

3.1.6 Subcategory: Not being lonely with the feeling

"FGD 4: I think so, because maybe it was only a year ago that I had some a-ha experience that 'Yes, but it is ... it is that.//…which I…where in'.// And then it felt somehow nice to know that, yes, but ... it is ... there are such concepts, it has happened to others..."

3.2 Category: Formal support

3.2.1 Subcategory: Lack of accessible formal support from the organization while there

"FGD 1: And then so it depends a bit... everyone was moving around all the time and was out in different places and like that ... there were usually some who stayed but there was nothing organized or so ... we had morning meetings but then you did not talk about such things but mora around what you would do during the day.//… So that, we probably had no such support ... but it was sort of your own."

S3. Additional file 2, Chosen excerpts

3.2.2 Subcategory: Formal support when home (debriefings, psychological support etc)

"FGD 2: P2. ... I really think, I am completely pro all kinds of psychologists, but I still think that it is a triumph to meet others who have been in the same situation or in similar or completely different situations..P1: Like, you two ..? P2: Yeah exactly, it would have been great to meet some of you ...

3.2.3 Subcategory: Lack of appropriate support when home

"FGD3: In (organization x) they have homecoming seminars, and so, then you can talk to others who have been in different places; you meet and it is great because you ... it is like, here that you exchange, like, experiences from different places and then relate to it. A bit like that, so I still think that. Talking about it makes, after all, makes you see that it is not just you who has felt this way. Well, it's more that thing that... helps in different situations so..."

3.3 Category: Preparatory support

3.3.1 Subcategory: Preparations, courses and trainings

"FGD 2: …we had a lot of group work and then, like one is supposed to struggle and do group work with someone who lives in another country and this, and everyone had different ideas about everything and everyone had different experiences. But after that, then we had each other. And then we have met a lot."

3.3.2 Subcategory: Briefings and information before

"FGD 2: It was my first assignment. So ... you try to ... read in somewhat. But then it was not really what was in this description that I actually was doing at the time. Then, what I needed to know it didn't say. Thus, such concrete details. If I'm going to write a referral on someone, how do I do it? Where should I make copies? Who will sign? Very handy stuff."

3.3.3 Subcategory: Lack of adequate information before

"FGD 3: Neah, I think I looked them up myself somewhere. No, that has been extremely bad. Extremely bad with those parts. These project-specific hand-overs…"

3.4 Category: Other tools

3.4.1 Subcategory: Other tools used to relieve stress while there

"FGD 2: Yes, it was actually not that bad with relaxation, different relaxation tips ... such files like this. Yes, like this: find a nice place, think of a nice place which you enjoy and then you got to, you should lie down and. And when I tried that. It was great ..."

3.4.2 Subcategory: Going back to work or take a pause

"FGD 2: It was: -Now, we will talk about all the investigations we're going to do and //, so. And I worked on it, and then finally came, one afternoon the janitor came in (saying) like this: How are things really with you? How are you? Then it was the janitor, not a close colleague. - You're not quite normal, are you all right? Here, it was so nice when someone just said that ..."

3.4.3 Subcategory: Own methods used to resolve/adopt when coming home

"FGD 1: What has been good for me, it has been to ... lecture. // Like in networks and like ... interest organizations and ... and schools and so. // ... So that ... it helped me through that in some way ... I think."

4 Theme 4: Engagement as a protective factor

4.1 Category: Altruistic motivation

4.1.1 Subcategory: Achieve good for others

"FGD 2: ... what really ... happens because there were so many television pictures and ... that ... there was so much inaccuracy I also thought ... about these things, that it was ...then I felt a bit more that I, but that it was a bit my duty to tell as well, about these fates and a little about how it really is and ... so..."

S3. Additional file 2, Chosen excerpts

4.1.2 Subcategory: Making a difference

"FGD 1: But then there is something that you can think that it is exciting and that you think it is ... is fun but somewhere you will still do your best or do what you think or somehow that is what makes the difference."

4.2 Category: Professional and personal motivation

4.2.1 Subcategory: Creating learning and experience

"FGD 1: Seems to be like ... almost all people who go away ... That there is more than one assignment ... So, it points towards that ... there is something ... in it for ... // something in it for personal development."

4.2.2 Subcategory: You do what you can

"FGD 3: But I can agree with that, despite being very unprepared, but I also felt that I was very ready with: 'You can do this'. It is not your fault that things are not in place, that no one has checked, really, it is not your fault. You do what you can, and… And that I think you get with age, like that security…"

4.3 Category: Framed by the professional role

4.3.1 Subcategory: Working within a structure

"FGD 3. After all, it has to do with structures, cultures, more than the care itself. Because I buy that… the first paper I signed was that I would follow the organization's guidelines and… I have accepted that. That I can't do what I do here at home, there. But, on the other hand, one can have a humanistic attitude about what one does. That it is not a yes or a no, but one can always give good care and see that this is a human being, like..."

4.3.2 Subcategory: You must follow guidelines

"FGD 1: According to the principles as well ... guidelines and ... there are often guidelines ... // if you talk about surgery or what you are talking about, antibiotics or whatever it is. // ... So that ... all employees then follow those. // ... That there is some structure with leadership and such, which gives some kind of control."

4.4 Category: Challenges are part of the work

4.4.1 Subcategory: We have problems in our own health care system

"FGD3: Yes, it is dilemmas, hospital beds and everyone who quits and how people leave for staffing agencies, and that costs us a lot of money instead of investing in those who are there and invest in nurses, in a just and decent working environment. It is horrible, it is really obnoxious when you work such shifts, I cannot cope with that ..."

4.4.2 Subcategory: Have agreed on the conditions

"FGD 3: It's like you go out and accept the medical ... part, that I cannot save a premature baby in (country x) even though she breathes, looks rosy and is alert. But I can give dignified care, worthy care."

5 Theme 5: Work environment stressors as a risk factor

5.1 Category: Work environment

5.1.1 Subcategory: Risk-taking in dangerous environments; security risk

"FGD 2: But that is a frustration that is quite common. In our assignments, people are hindered from travel because it is so difficult… so special ... those who go to Syria now and away to Iraq ... // So maybe you end up outside (the country) and are in some hotel ... at some strange place nearby ... and people tend to feel quite bad about that…"

5.1.2 Subcategory: Taking a risk of not being able to go back to normal work directly; financial and personal health risk

"FGD 2: P1. It can be beautiful. When I see the pictures now, I see it, but didn't I see how beautiful it was? But I don't think I did. Because it was so difficult. P2: It was so, it was really tough. But it was because when I was there, it was so unsettled too, so you felt that ... everywhere it was turbulent, and you dared not go outside ..."

S3. Additional file 2, Chosen excerpts

5.1.3 Subcategory: Traumatic events

"FGD 2: Nah, but, but I think it was so much more concrete. // I mean a robbery and weapon, this all can be understood, and then they certainly have some PM then, because it has certainly happened, eh... But like this other, with which you go in this, uh, this reality every single day…"

5.2 Category: Stress and exhaustion

5.2.1 Subcategory: Malfunctioning team

"FGD 1: While it can also be a problem or, if it is ... if you are in such a small place or like a small village with a fence around and ... some huts ... where you live and then // ... the problem itself is one in the group (the team) or so ... then it can also be ... on the contrary then, or those who drank (alcohol) very much or ... It could be things like that too ... maybe the opposite that it was difficult to ... be able to bring up these things as well. "

5.2.2 Subcategory: Facing continuous suffering

"FGD 4: … That's what has been the most difficult… it was so difficult there and then, because there was no time to… reflect… //… like… we had… it was an emergency assignment where you were out… on the boat like fifteen hours a day and then go home and prepare for the next day… // and then up and go to the next and… there was someone… new as well… similar story that you met… so it was like…//… It was not so easy to handle it there and then you only got to … // ... push forward"

5.2.3 Subcategory: Having a role of responsibility in stressful environments

"FGD 2: Pregnant women ... with small children and rubber boats and ... only that, you get so influenced by how miserable...how horrible people have it and that they still have the power to ... take this journey ... So yeah … it is also that you are weakened by...just by the situation ... Emotionally within the situation, in many situations…//...you see so much suffering...which might weaken you...already before a specific event happens… I can think …"
